# Combined Mutations of LTBP3 and COL5A1A in Geleophysic Dysplasia

**DOI:** 10.1155/carm/1910227

**Published:** 2026-06-10

**Authors:** Adel Alsharei, Mohammad T. Batayneh, Firas Zraiqi, Yazan M. Al-Omari, Abdullah M. Al-Ali, Almu’atasim Khamees, Saleh A. Ba-shammakh, Aiman Al Sharei, Raed M. Al-Zoubi, Mazhar S. Al Zoubi

**Affiliations:** ^1^ Faculty of Medicine, Yarmouk University, P.O. Box 566, Irbid, 21163, Jordan, yu.edu.jo; ^2^ Princess Basma Teaching Hospital, Ministry of Health, P.O. Box 63001, Irbid, 22110, Jordan, moh.gov.jo; ^3^ Department of Pharmacology, Community Medicine and Clinical Skills, Faculty of Medicine, The Hashemite University, Al Zarqa, Jordan, hu.edu.jo; ^4^ Surgical Research Section, Department of Surgery, Hamad Medical Corporation, Doha, Qatar, hamad.qa; ^5^ Department of Chemistry, Jordan University of Science and Technology, Irbid, 22110, Jordan, just.edu.jo; ^6^ Department of Biomedical Sciences, QU-Health, College of Health Sciences, Qatar University, Doha, 2713, Qatar, qu.edu.qa; ^7^ Department of Basic Medical Sciences, Faculty of Medicine, Yarmouk University, Irbid, 21163, Jordan, yu.edu.jo

**Keywords:** bone fracture, COL5A1, geleophysic dysplasia, LTBP3

## Abstract

Geleophysic dysplasias (GDs) are uncommon genetically predisposed abnormalities that interfere with skeletal growth and formation. Several GD subtypes have different clinical manifestations. The current report presents the case of a 7‐year‐old Syrian boy with a medical history of repeated bone fractures and noticeable facial characteristics. Initial laboratory examinations revealed normal results, except for low serum phosphate and ferritin levels. The X‐ray images showed no abnormalities. The DEXA scan was like that of a 92.1‐year‐old. Karyotype analysis revealed 46 XY. Genetic testing results showed compound heterozygous mutations in the Latent Transforming Growth Factor Beta Binding Protein 3 (LTBP3) gene and a heterozygous mutation in collagen Type V Alpha 1 (COL5A1), consistent with the patient’s clinical manifestations. The following fractures were treated using a combination of nonsurgical casting and surgical intervention. The LTBP3 gene mutations are associated with GD. However, the COL5A1 gene mutations are assumed to be associated with Ehlers–Danlos syndrome Type 1. However, the patient did not exhibit the typical features of joint hypermobility or skin abnormalities, suggesting that the COL5A1 mutation may have a minor effect on his condition. In conclusion, this case highlights the importance of genetic testing in children with repeated fractures and abnormal physical traits. Although the COL5A1 mutation may have a minor influence, further investigation is needed to understand its long‐term effects. This instance also emphasizes the range of physical characteristics that can occur with GD and Marfan syndrome, even when caused by the same genetic mutations.

## 1. Introduction

The development of the skeleton is a complex process involving the interaction of different cellular components within the growth plates, also known as epiphyses [1]. Epiphyses are specialized regions at the ends of long bones [2]. Geleophysic dysplasias (GDs) are rare bone disorders attributed to genetic abnormalities that interfere with this intricately orchestrated process [3]. Moreover, GDs are associated with multiple syndromes and various genetic predispositions, which increases the burden on patients and their families. In addition, the clinical diagnosis and management of these cases are challenging.

A wide variety of skeletal illnesses fall under the umbrella of GDs [4]. Each of these disorders has distinct clinical characteristics and underlying genetic causes. Short stature, joint pain, and limited movement are the most common symptoms associated with multiple epiphyseal dysplasia (MED) [5], the most common variant. However, the clinical picture can vary widely among the different GD subtypes, with some patients exhibiting severe bone abnormalities, early onset osteoarthritis, and limited mobility. In contrast, others present milder phenotypes with few functional restrictions [6].

Significant advancements have been made in elucidating the genetic landscape of GDs. These advancements have revealed that mutations in genes encoding essential structural components of the growth plate [7], such as Type X Collagen Alpha 1 (COL10A1) in MED, disrupt the intricate architecture of the extracellular matrix (ECM), thereby impairing normal chondrocyte function and ultimately affecting bone growth. Mutations in genes involved in growth factor signaling pathways and other cellular processes important for growth plate development have been linked to various GD subtypes [7].

In addition, studies have shown that some environmental exposures, such as nutritional inadequacies or teratogenic insults during fetal development, may interact with genetic predisposition, potentially influencing the severity of the disease or the way it manifests [8]. Therefore, determining how genetic and environmental variables interact offers great potential for developing a more comprehensive knowledge of the pathophysiology of GD.

At the molecular level, various studies have demonstrated that GD represents a genetically heterogeneous group of rare disorders characterized by distinctive facial features and progressive joint limitation [9, 10]. Specifically, certain genetic subtypes have been identified: GD1, GD2, and GD3, which are associated with biallelic, A Disintegrin and Metalloproteinase with Thrombospondin Motifs‐Like 2 (ADAMTSL2) mutations, heterozygous Fibrillin‐1 (FBN1) mutations, and LTBP3 mutations, respectively. Notably, these subtypes share disrupted transforming growth factor beta (TGF‐β) signaling as a common pathogenic mechanism [11–15]. LTBP3 is a critical ECM protein that regulates TGF‐β bioavailability and skeletal development [16, 17]. Consequently, disruption of LTBP3 function can lead to dysregulated TGF‐β signaling, which may be linked to skeletal abnormalities—a hallmark feature of GD [18, 19]. This molecular convergence, combined with phenotypic variability, underscores the vital importance of comprehensive genetic testing for accurate diagnosis and management [20]. Additionally, cell trafficking disorders have been suggested to contribute to the pathogenesis of skeletal dysplasia, highlighting the significance of this mechanism in the development of GD [21].

Currently, treatment and cure of GDs are not available [22]. However, management options generally focus on reducing symptoms, enhancing mobility, and preventing complications [23]. Physical therapy is an essential component of preserving joint flexibility and strength [24]. Orthotics and braces are devices that can assist in correcting or supporting skeletal abnormalities [25]. In extreme circumstances, surgical procedures may be required to correct bone abnormalities or alleviate osteoarthritis symptoms [25]. This case study aims to demonstrate a case of a child with GD.

A pediatric patient with GD due to compound heterozygous LTBP3 mutations and a concurrent COL5A1 variant, highlighting the clinical presentation, genetic findings, and management approach to expand the phenotypic spectrum of this rare disorder, was reported.

## 2. Clinical Presentation

The current case is of a 7‐year‐old Syrian boy with a medical history of repeated bone fractures and noticeable facial characteristics. The investigation revealed that the patient’s sister was also affected, presenting with similar clinical features of GD. However, no other family members, including the parents, exhibited any signs or symptoms suggestive of GD or related connective tissue disorders. The patient was initially presented to the clinic at the age of 5 years after sustaining a simple fracture of the right proximal tibia due to a minor injury. The patient’s condition was managed with a cast. Over the next 2 months, the patient suffered two further nondisplaced fractures. One occurred in the middle section of his left tibia, while the other was a fracture in the middle of his left tibia without bending or angulation. The patient was subjected to conservative treatment involving the application of casts. However, one week after the cast was removed from the third fracture, the patient experienced a fall from a low height (50 cm). He had a right tibial midshaft fracture with anterior angulation. A cast was necessary for this fracture because of the angulation. Physical examination revealed additional dysmorphic features, including a “happy” facial expression, prominent cheeks, a broad nasal bridge, elongated eyelashes, and unusually fragile teeth. However, the examination was unremarkable (Figure 1). An echocardiogram was performed at the age of 7 years. The examination showed that the cardiac muscle and heart valves were functioning normally, which is a positive indication that there are no potential issues related to GD. The following fractures were treated using a combination of non‐surgical casting (left tibia) and surgical intervention with an elastic stable intramedullary nail (right tibia) (Figure 2). However, due to recurrent fractures with angulation deformity, a 3‐point compression orthosis was applied for the right leg (Figure 3). Vitamin D and K2 supplementation started.

**Figure FIGURE 1:**
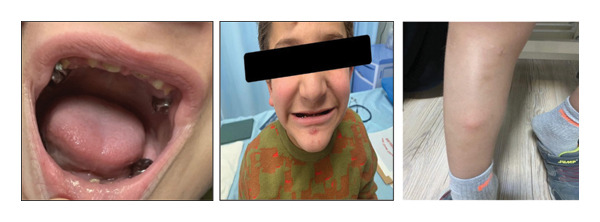
Clinical photograph of the patient at age 7. The image demonstrates characteristic dysmorphic facial features associated with geleophysic dysplasia, including a “happy” facial expression with full cheeks, a broad nasal bridge, and elongated eyelashes. Note the absence of overt skin abnormalities or joint hypermobility.

**Figure FIGURE 2:**
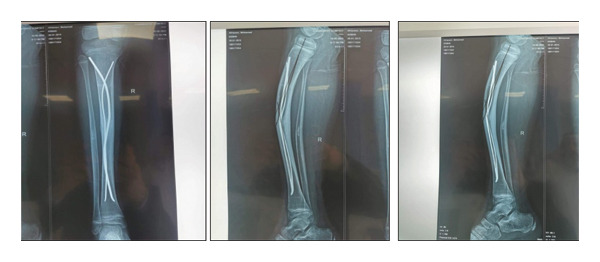
Postoperative radiograph demonstrating an elastic stable intramedullary nail (ESIN) in the right tibia following surgical fixation for the midshaft fracture with anterior angulation.

**Figure FIGURE 3:**
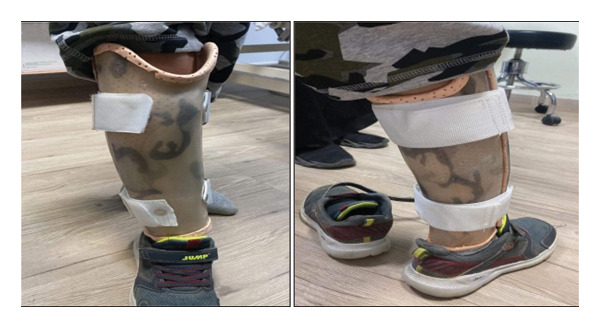
Application of a 3_point compression orthosis to the right leg to correct anterior angulation of the tibial midshaft fracture following recurrent fracture after elastic stable intramedullary nail (ESIN) fixation.

Radiographs of the long bones, skull, chest, and pelvis showed no abnormalities or clear indications of osteogenesis imperfecta. Nevertheless, both legs had ongoing repeated fractures. The patient was referred to a dentist because of significant dental caries affecting all primary teeth and partially erupted permanent molars (Figure 4). The dentist identified a condition of reduced mineralization affecting both primary and permanent teeth, accompanied by several abscesses that were subsequently treated. The DEXA scan results indicated a Z‐score of 0.2, which is within the normal range for someone 92.1 years old.

**Figure FIGURE 4:**
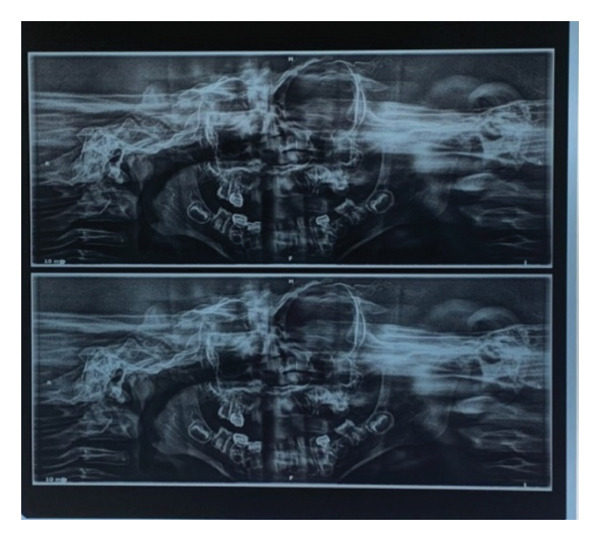
Intraoral photograph demonstrating severe dental manifestations. The image shows significant dental pathology, including extensive caries affecting all primary teeth, hypomineralization, and partially erupted permanent molars. Multiple periapical abscesses are present, consistent with the dental fragility observed in this connective tissue disorder.

Initial laboratory studies showed normal results for thyroid function tests, total blood count, kidney function tests, liver function tests, and Vitamin D levels. Nevertheless, low serum phosphate and ferritin levels were detected. These inadequacies were rectified by administering oral phosphate and iron supplements. Karyotype analysis showed that the individual was a normal male with 46 chromosomes, including XY sex chromosomes. MRI examination revealed that the pituitary gland and sella turcica were normal.

Whole‐exome sequencing is performed on genomic DNA using the Agilent SureSelect Clinical Research Exome v3 targeted sequence capture method to enrich for the exome. Direct sequencing of the amplified captured regions was performed using 2 × 150 bp reads on Illumina next‐generation sequencing (NGS) systems. Primary data analysis is performed using Illumina bcl2fastq converter v2.19. Secondary analysis is performed using Illumina DRAGEN Bio‐IT Platform v3.4.12. Tertiary data analysis is performed using SnpEff v4.31 and PerkinElmer’s internal ODIN v.1.01 software. CNV and the absence of heterozygosity are assessed using BioDiscovery’s NxClinical v5.1 software. Genetic testing results showed the presence of compound homozygous mutations. The LTBP3 gene showed a homozygous nonsense variant, namely, c.3574G > T (p.Glu1192∗) nonsense mutation. The COL5A1 gene harbored a heterozygous missense mutation, specifically c.4172A > G (p.Lys1391Arg). The identified mutations provided a genetic foundation for the clinical manifestations in patients. The LTBP3 gene mutation is linked to GD. This connective tissue disease is characterized by short stature, unique facial features, skeletal abnormalities, and the possibility of heart valve, lung, and liver complications. This mutation likely interferes with TGF‐β signaling, a critical pathway for proper development [13, 26]. COL5A1 gene mutation refers to a change or alteration in the COL5A1 gene. COL5A1 genetic alterations are linked to Ehlers–Danlos syndrome (EDS) Type 1, characterized by excessive joint mobility, delicate skin, and a tendency to bruise easily [27]. Nevertheless, the patient did not exhibit the usual excessive flexibility in the joints and skin characteristics associated with EDS Type 1, indicating that the COL5A1 mutation (p.Lys1391Arg) may have a less significant (benign) impact on his condition which is supported by the NCBI according to the American College of Medical Genetics and Genomics–Association for Molecular Pathology (ACMG–AMP). However, according to the ACMG/AMP 2015 guidelines, we found that the LTBP3 c.3574G > T (p.Glu1192∗) variant was classified as pathogenic based on PVS1, PM2, and PP4. The COL5A1 c.4172A > G (p.Lys1391Arg) variant was classified as likely pathogenic upon adoption of PM1, PM5, PP2, PP3, and PP4. A detailed justification for these classifications is provided in the text [28].

## 3. Discussion

The homozygous nonsense mutation (c.3574G > T) in the LTBP3 gene has been linked to GD, a rare connective tissue disorder [29]. This disorder is characterized by short stature, specific facial features (such as a round face, full cheeks, and broad nasal bridge), and skeletal abnormalities (including repeated fractures) [15], and potential complications affecting the heart valves, lungs, and liver [28]. The current case presented multiple features of GD, including a distinctive “happy” facial expression, recurrent bone fractures, and normal echocardiographic findings (except for possible future problems). This case contributes to the expanding body of research that establishes a connection between LTBP3 mutations, GD, and related clinical symptoms.

The presence of a heterozygous missense mutation (c.4172A > G) in the COL5A1 gene is assumed to be linked with EDS Type 1 [30], which is characterized by excessive joint mobility, delicate skin, and a tendency to bruise easily [31]. Nevertheless, our patient did not exhibit the usual characteristics of joint hypermobility and skin abnormalities associated with EDS Type 1 [32]. This indicates that the COL5A1 mutation may have a minor influence on his condition, possibly functioning as a modifier or adding to the general weakness of the skeletal system [33]. Additional research is required to comprehend the enduring consequences of this mutation and its potential interplay with LTBP3 mutations.

Table 1 summarizes our patient’s condition compared with previously documented cases of GD (associated with FBN1 mutations) and Marfan syndrome (associated with FBN1 mutations). Although there are some similarities in physical traits, such as short stature and distinct facial features (broad nose and wide bridge), our case shows a less severe manifestation than others. This emphasizes the range of physical characteristics observed in individuals with various genetic diseases, even with the same mutations.

**Table TABLE 1:** Summary of the clinicopathological information of the current case of geleophysic dysplasia (GD) compared to previous case studies.

Case	Age at presentation	Gender	Gene defect	Clinical findings and location of the disease	Management
Our case	7 years old	Male	Mutations in LTBP3 and COL5A1	➢ Transverse right proximal tibial fracture ➢ Left mid‐tibial fracture ➢ ”happy” face with full cheeks, broad long bridge of the nose, long eyelashes, and abnormal weakness of the teeth ➢ His height is 130.0 cm ➢ Normal thyroid hormone, hematology, KFT, LFT, and Vitamin D levels. ➢ Low phosphate and ferritin levels ➢ Echocardiography showed normal cardiac muscle and normal heart valves.	Vitamin D 2000 IU daily Vitamin K2 Leg 360̊ plastic brace with 3‐point antecurvatum correction

Case 1 [29]	At the age of 3 years and 8 months	Male	Two heterozygous mutations in the ADAMTSL2 gene	➢ Severe short stature, atrophy of the limbs and hands, joint stiffness, shortened fingers and toes, rough and thick skin, short nose, and a round face with full cheeks and a flat nose bridge. ➢ Thinning of the upper lip, philtrum, and a high, sharp voice ➢ Small ears, short metatarsals, short neck, narrow heart valve with thickening of the wall, and clearly visible in the mitral and aortic valves, no recurrent inflammation or liver enlargement appeared.	Subcutaneous injection of human growth hormone in a combined dose (0.15 units per kilogram of body weight daily).

Case 2 [30]	10 years and 2 months	Male	Heterozygous missense mutation in the FBN1 gene	➢ Low arm span (−3.4 SDS) ➢ Asymmetry in height and extremely short stature, a small hand length of 14.4 cm with small hand size and short fingers, as the middle finger reached 6.4 cm (3%), and a short palm with a length of only 8 cm. ➢ With a large nose, a bulging bridge, and relatively large lips with a prominent philtrum, there are no other obvious facial deformities. ➢ The skin appears natural in appearance and has a natural thickness. ➢ There are no prominent eyebrows, a natural mouth size, and no long eyelashes, but it is completely natural. ➢ When the actual age was 10 years and 2 months, the bone age was approximately 3‐4 years less.	Human growth hormone in a combined dose (50–60 μg per kilogram of body weight daily).

Case 3 [30]	7 years	Female	Heterozygous missense mutation in the FBN1 gene	➢ Wide nose bridge, slight spacing, thick lips. ➢ Severe short stature (SDS‐4) ➢ There are no deformities in the muscles or bones, the skin is normal, the walking movement is normal, and there are only small hands and feet. ➢ An echocardiogram showed that the heart was free of valvular or structural defects. ➢ The age of the bones was only about 2 years less.	Not available

Case 4 [31]	7 years and 6 months	Male	Heterogeneity in mutations in the FBN1 gene	➢ Eyes well apart, short neck, flat nose, round face, concave chest, extended elbow joint. ➢ The liver appears about 3 cm below the edge of the right ribs, while the spleen is not clearly visible. ➢ A diminished holosystolic murmur of Grades 4‐5 was evident in the precordium. ➢ Reduced bone age (age was about 3 years). ➢ At the age of 12, the symptoms were reassessed, and a clear bilateral elbow deformity was observed with active finger movement. ➢ Ultrasound of the heart revealed enlargement of the right ventricle with a normal size of the right atrium. Tricuspid regurgitation also appeared to a small extent, but without regurgitation or pulmonary stenosis. ➢ The location of the liver was clear and fixed, about 8 cm below the right of the costal margin of the spleen, but the spleen was not palpable. ➢ The thoracolumbar distance increased from the intervertebral foramen, and oval‐shaped bodies appeared in the lateral spine radiograph.	Not available

Case 5 [31]	3 years and 9 months	Female	Heterogeneity in mutations in the FBN1 gene	➢ She had short stature and hepatosplenomegaly ➢ It was found that she suffers from stenosis of the pulmonary valves and slight tricuspid regurgitation. ➢ Her growth retardation was remarkable 1 year after birth. ➢ Short upturned nose, flat and long philtrum, “happy” face with narrow and short eyelids, plump cheeks, liver appeared 7 cm below the right costal margin, while the spleen was 1.5 cm below the left costal margin. ➢ The bone age was estimated at 1.5 years, while the actual age was 2.5 years.	Not available

*Note:* ADAMTSL2, A Disintegrin and Metalloproteinase with Thrombospondin Motifs‐Like 2; FBN1, Fibrillin 1; LTBP3, Latent Transforming Growth Factor Beta Binding Protein 3; COL5A1, Collagen Type V Alpha 1 Chain.

Abbreviations: ESIN, elastic stable intramedullary nail; KFT, kidney function test; LFT, liver function test.

In our case, there were combined mutations in the LTBP3 and COL5A1 genes. However, other reported cases of a heterozygous missense mutation in the FBN1 gene (Cases 2–5 in the table) [34, 35], while Case 1 reported two heterozygous mutations in the ADAMTSL2 gene [30]. In addition, almost all reported cases have short stature, small hand length, small hand size, and short fingers [30, 34, 35]. Our patient had a normal echocardiogram with normal cardiac muscle and heart valves, consistent with the findings of Case 3 [34]. A narrow heart valve with wall thickening clearly visible in the mitral and aortic valves was observed in Case 1 [19], and a holosystolic murmur of Grades 4‐5 and enlargement of the right ventricle and tricuspid regurgitation were observed in Case 4 [35]. In addition, echocardiography in Case 5 revealed stenosis of the pulmonary valves and slight tricuspid regurgitation [35].

The skin appeared normal and had a natural thickness in our case, which is consistent with the findings of Cases 2 and 3 [34], while the skin was rough and thick in Case 1 [30]. Moreover, most patients had normal liver function tests and normal liver and spleen sizes, except for Case 5, which revealed hepatosplenomegaly [35].

There is a special facial characteristic for patients mentioned in our case who had “happy” facial expressions, prominent cheeks, a broad nasal bridge, and elongated eyelashes, which was similar for Cases 4 and 5 [35]. Also, they may have a thin upper lip philtrum, as in Cases 1 [30], while large lips and a prominent philtrum, as in Cases 2 and 3 [34]. The current results showed the spectrum of genotypic and phenotypic syndromes associated with GPs, underscoring the importance of genetic testing for children who experience recurrent fractures and exhibit abnormal physical traits and facial dysmorphic features.

At the molecular level, the LTBP3 isoform is a crucial ECM protein known to interact with FBN1 [18]. In addition, LTBP3 showed a role in the TGFβ signaling that is involved in bone formation and cardiovascular tissues in animal models [11–13, 19, 36]. For instance, the nonsense mutation Y744X has been reported in short‐stature and oligodontia cases [16, 18, 37]. The detected nonsense mutation in the current study will result in a truncated, nonfunctional LTBP3, affecting the normal ECM composition in various tissues, including the bones. Therefore, functional studies of this mutation (p.Glu1192∗) reveal its molecular impact on GP development. The available data describe the impact of LTBP3 knockout on skeletal system development in mice [36]. On the other hand, the detected mutation in the COL5A1 gene defect is expected to be associated with the EDS syndrome; however, the detected mutation is a point mutation that alters Lys to Arg (p.Lys1391Arg), which is preferred to be a benign genetic alteration according to the ACMG–AMP [28]. However, functional studies are recommended to determine the impact of this point mutation on collagen tTpe V function.

This case study is constrained by the limited accessibility of data pertaining to a solitary patient. Long‐term surveillance is essential to monitor potential consequences of GD on cardiovascular, respiratory, and hepatic function. Furthermore, conducting functional tests to evaluate joint mobility could offer additional insights into the potential impact of the COL5A1 mutation. Generally, the major limitation of the diagnosis of this disease is its rarity, which hinders genetic investigation; therefore, the current study underscores the significance of clinical investigation of GPs in the region. In addition, further follow‐up may be discontinued due to various reasons, such as relocation of the patients.

This instance highlights the significance of taking genetic testing into account for children who experience repeated fractures and have abnormal physical traits. The mutations found in the LTBP3 and COL5A1 genes explain the hereditary cause of the patient’s weakened bones and unique facial characteristics. Although the COL5A1 mutation may have a minor influence, further investigation is needed to fully understand its long‐term consequences. This instance also emphasizes the range of physical characteristics that can occur with GD and Marfan syndrome, even when caused by the same genetic mutation. The primary objective of ongoing care is to proactively avoid fractures, optimize bone health, and closely monitor any problems related to GD. In addition, this case expands the phenotypic spectrum of LTBP3‐related GD and underscores the importance of comprehensive genetic testing in children with recurrent fractures and dysmorphic features. The concurrent COL5A1 variant may represent a modifier, though further investigation is needed. Multidisciplinary management focusing on fracture prevention and surveillance for systemic complications is essential.

## Author Contributions

Mazhar S. Al Zoubi, Raed M. Al‐Zoubi, Almu’atasim Khamees, and Mohammad T. Batayneh: conceptualization, methodology, investigation, data curation, writing–original draft, and writing–review and editing. Firas Zraiqi, Yazan M. Al‐Omari, Abdullah M. Al‐Ali, and Saleh A. Ba‐shammakh: methodology, investigation, and data curation. Mazhar S. Al Zoubi and Raed M. Al‐Zoubi: methodology and writing–review and editing the final draft.

## Funding

No funding was received for this research.

## Disclosure

The work has been reported in line with the SCARE criteria [38].

This case report is not eligible for obtaining a research registry since it only contains a report of a known entity with no new surgical or medical interventions.

## Ethics Statement

No ethical approval is required.

## Consent

Written informed consent was obtained from the patient’s father for publication and any accompanying images. A copy of the written consent form is available for review by the editor‐in‐chief of this journal upon request.

## Conflicts of Interest

The authors declare no conflicts of interest.

## Data Availability

The data that support the findings of this study are available from the corresponding author upon reasonable request.

## References

[1] Provot S. , Schipani E. , Wu J. , and Kronenberg H. , Marcus and Feldman’s Osteoporosis, 2021, Elsevier, 39–73, Development of the Skeleton.

[2] Tran N. T. , Kim Y.-K. , Kim S. Y. , Lee M. H. , and Lee K. B. , Comparative Osteogenesis and Degradation Behavior of Magnesium Implant in Epiphysis and Diaphysis of the Long Bone in the Rat Model, Materials. (2022) 15, no. 16, 10.3390/ma15165630. PMC9416751 36013766

[3] Markova T. , Kenis V. , Melchenko E. et al., Clinical and Genetic Characteristics of Multiple Epiphyseal Dysplasia Type 4, Genes. (2022) 13, no. 9, 10.3390/genes13091512. PMC9498659 36140680

[4] Handa A. , Nishimura G. , Zhan M. X. , Bennett D. L. , and El-Khoury G. Y. , A Primer on Skeletal Dysplasias, Japanese Journal of Radiology. (2022) 40, no. 3, 245–261, 10.1007/s11604-021-01206-5. 34693503 PMC8891206

[5] Lv S. , Zhao J. , Liu L. et al., Exploring and Expanding the Phenotype and Genotype Diversity in Seven Chinese Families With Spondylo-Epi-Metaphyseal Dysplasia, Frontiers in Genetics. (2022) 13, 10.3389/fgene.2022.960504. PMC9473317 36118854

[6] Kizilkaya V. , Engin S. , Tunc A. , and Tonbul A. , Multiple Epiphyseal Dysplasia Tip 5: Case Report a Rare Skeletal Dysplasıa Presenting With Repetitive Joint Pain in Children, International Journal of Surgery Case Reports. (2023) 106, 10.1016/j.ijscr.2023.108179. PMC10139900 37062195

[7] Ramzan F. , Salim A. , and Khan I. , Cartilage: From Biology to Biofabrication, 2023, Springer, 125–154, Signaling Pathways Regulating Cartilage Formation.

[8] Gómez-Roig M. , Pascal R. , Cahuana M. et al., Environmental Exposure During Pregnancy: Influence on Prenatal Development and Early Life: A Comprehensive Review, Fetal Diagnosis and Therapy. (2021) 48, no. 4, 1–13, 10.1159/000514884. 33735860

[9] Huckert M. , Stoetzel C. , Morkmued S. et al., Mutations in the Latent TGF-Beta Binding Protein 3 (LTBP3) Gene Cause Brachyolmia With Amelogenesis Imperfecta, Human Molecular Genetics. (2015) 24, no. 11, 3038–3049, 10.1093/hmg/ddv053. 25669657 PMC4424950

[10] Rifkin D. B. , Rifkin W. J. , and Zilberberg L. J. M. B. , LTBPs in Biology and Medicine: LTBP diseases, 2018, 71, 90–99. 10.1016/j.matbio.2017.11.014 PMC5988920 29217273

[11] Zhou Y. , Cashman T. J. , Nevis K. R. et al., Latent TGF-β Binding Protein 3 Identifies a Second Heart Field in Zebrafish, Nature. (2011) 474, no. 7353, 645–648, 10.1038/nature10094. 21623370 PMC3319150

[12] Spranger J. , Gilbert E. , Tuffli G. , Rossiter F. , and Opitz J. J. T. L. , Geleophysic Dwarfism—A Focal Mucopolysaccharidosis?, The Lancet. (1971) 298, no. 7715, 97–98, 10.1016/s0140-6736(71)92073-3. 4104008

[13] Marzin P. , Thierry B. , Dancasius A. et al., Geleophysic and Acromicric Dysplasias: Natural History, Genotype–Phenotype Correlations, and Management Guidelines From 38 Cases, Genetics in Medicine. (2021) 23, no. 2, 331–340, 10.1038/s41436-020-00994-x. 33082559

[14] Le Goff C. , Morice-Picard F. , Dagoneau N. et al., ADAMTSL2 Mutations in Geleophysic Dysplasia Demonstrate a Role for ADAMTS-Like Proteins in TGF-β Bioavailability Regulation, Nature Genetics. (2008) 40, no. 9, 1119–1123, 10.1038/ng.199. 18677313 PMC2675613

[15] Allali S. , Le Goff C. , Pressac–Diebold I. et al., Molecular Screening of ADAMTSL2 Gene in 33 Patients Reveals the Genetic Heterogeneity of Geleophysic Dysplasia, Journal of Medical Genetics. (2011) 48, no. 6, 417–421, 10.1136/jmg.2010.087544. 21415077 PMC4413937

[16] Robertson I. B. , Horiguchi M. , Zilberberg L. , Dabovic B. , Hadjiolova K. , and Rifkin D. B. J. M. B. , Latent TGF-β-binding Proteins, Matrix Biology: Journal of the International Society for Matrix Biology. (2015) 47, 44–53, 10.1016/j.matbio.2015.05.005. 25960419 PMC4844006

[17] Dabovic B. , Chen Y. , Colarossi C. et al., Bone Abnormalities in Latent TGF-β Binding Protein (Ltbp)-3–Null Mice Indicate a Role for Ltbp-3 in Modulating TGF-β Bioavailability, The Journal of Cell Biology. (2002) 156, no. 2, 227–232, 10.1083/jcb.200111080. 11790802 PMC2199217

[18] Koli K. , Ryynänen M. J. , and Keski-Oja J. J. B. , Latent TGF-β Binding Proteins (LTBPs)-1 and-3 Coordinate Proliferation and Osteogenic Differentiation of Human Mesenchymal, Stem Cells. (2008) 43, no. 4, 679–688, 10.1016/j.bone.2008.06.016. 18672106

[19] Chen G. , Deng C. , Li Y. , and Pjijobs , TGF-β and BMP Signaling in Osteoblast Differentiation and Bone Formation, International Journal of Biological Sciences. (2012) 8, no. 2, 272–288, 10.7150/ijbs.2929. 22298955 PMC3269610

[20] Le Goff C. , Mahaut C. , Wang L. W. et al., Mutations in the TGFβ Binding-Protein-Like Domain 5 of FBN1 are Responsible for Acromicric and Geleophysic Dysplasias, The American Journal of Human Genetics. (2011) 89, no. 1, 7–14, 10.1016/j.ajhg.2011.05.012. 21683322 PMC3135800

[21] BjtaoP T. , 2025, 60, no. 6, Cell Trafficking Disorders Play an Important Role in the Pathogenesis of Skeletal Dysplasias. 10.5152/TurkArchPediatr.2025.25159 PMC12613505 41257398

[22] Sananta P. , Lesmana A. , and Alwy Sugiarto M. , Growth Plate Injury in Children: Review of Literature on PubMed, Journal of Public Health Resources. (2022) 11, no. 3, 10.1177/22799036221104155. PMC9340334 35923296

[23] Moy N. , Flynn D. , Henriquez J. , Connelly L. B. , Vale L. , and Paolucci F. , Interventions for Improving Clinical Outcomes and Health-Related Quality-of-Life for People Living With Skeletal Dysplasias: an Evidence Gap Map, Quality of Life Research. (2023) 32, no. 10, 2751–2762, 10.1007/s11136-023-03431-z. 37294397 PMC10474209

[24] Chang Y. Y. , Lee C. C. , Lin S. C. , Kuo K. N. , Wu K. W. , and Wang T. M. , 2021, The Early Outcome of the Bernese Periacetabular Osteotomy for Hip Dysplasia in Multiple Epiphyseal Dysplasia. 10.1186/s13023-023-02920-1 PMC10614309 37904148

[25] Brown R. and Monsell F. , Understanding the Skeletal Dysplasias, Current Orthopaedics. (2003) 17, no. 1, 44–55, 10.1054/cuor.2002.0307.

[26] Legare J. M. , Modaff P. , Strom S. P. , Pauli R. M. , and HljajoMGPA B. , Geleophysic Dysplasia: 48 Year Clinical Update with Emphasis on Cardiac Care, American Journal of Medical Genetics, Part A. (2018) 176, no. 11, 2237–2242, 10.1002/ajmg.a.40377. 30195254

[27] Ritelli M. , Dordoni C. , Venturini M. et al., Clinical and Molecular Characterization of 40 Patients With Classic Ehlers–Danlos Syndrome: Identification of 18 COL5A1 and 2 COL5A2 Novel Mutations, Orphanet Journal of Rare Diseases. (2013) 8, no. 1, 10.1186/1750-1172-8-58. PMC3653713 23587214

[28] Nykamp K. , Anderson M. , Powers M. et al., Sherloc: A Comprehensive Refinement of the ACMG–AMP Variant Classification Criteria, Genetics in Medicine. (2017) 19, no. 10, 1105–1117, 10.1038/gim.2017.37. 28492532 PMC5632818

[29] Huckert M. , Stoetzel C. , Morkmued S. et al., Mutations in the Latent TGF-Beta Binding Protein 3 (LTBP3) Gene Cause Brachyolmia With Amelogenesis Imperfecta, Human Molecular Genetics. (2015) 24, no. 11, 3038–3049, 10.1093/hmg/ddv053. 25669657 PMC4424950

[30] Li D. , Dong H. , Zheng H. et al., A Chinese Boy With Geleophysic Dysplasia Caused by Compound Heterozygous Mutations in ADAMTSL2, European Journal of Medical Genetics. (2017) 60, no. 12, 685–689, 10.1016/j.ejmg.2017.09.003. 28917829

[31] Anjum H. M. , Ezhilarasi A. , Hannah J. C. , Kanagarani K. , and Chetty S. , 2020, Ehler Danlos Syndrome.

[32] Yew K. S. , Kamps-Schmitt K. A. , and Borge R. , Hypermobile Ehlers-Danlos Syndrome and Hypermobility Spectrum Disorders, American Family Physician. (2021) 103, no. 8, 481–492. 33856167

[33] Errichiello E. , Malara A. , Grimod G. , Avolio L. , Balduini A. , and Zuffardi O. , Low Penetrance COL5A1 Variants in a Young Patient With Intracranial Aneurysm and Very Mild Signs of Ehlers-Danlos syndrome, European Journal of Medical Genetics. (2021) 64, no. 1, 10.1016/j.ejmg.2020.104099. 33189937

[34] De Bruin C. , Finlayson C. , Funari M. F. et al., Two Patients With Severe Short Stature due to a FBN1 Mutation (P. Ala1728Val) With a Mild Form of Acromicric Dysplasia, Hormone Research in Paediatrícs. (2016) 86, no. 5, 342–348, 10.1159/000446476. 27245183 PMC5135661

[35] Wang Y. , Zhang H. , Ye J. , Han L. , and Gu X. , Three Novel Mutations of the FBN1 Gene in Chinese Children With Acromelic Dysplasia, Journal of Human Genetics. (2014) 59, no. 10, 563–567, 10.1038/jhg.2014.73. 25142510

[36] Dabovic B. , Chen Y. , Colarossi C. , Zambuto L. , Obata H. , and Rifkin D. , Beyond Carrier Proteins Bone Defects in Latent TGF-β Binding Protein (Ltbp)-3 Null Mice; A Role for Ltbp in TGF-β Presentation, Journal of Endocrinology. (2002) 175, no. 1, 129–141, 10.1677/joe.0.1750129. 12379497

[37] Noor A. , Windpassinger C. , Vitcu I. et al., Oligodontia is Caused by Mutation in LTBP3, the Gene Encoding Latent TGF-β Binding Protein 3, The American Journal of Human Genetics. (2009) 84, no. 4, 519–523, 10.1016/j.ajhg.2009.03.007. 19344874 PMC2667979

[38] Sohrabi C. , Mathew G. , Maria N. et al., The SCARE 2023 Guideline: Updating Consensus Surgical Case Report (SCARE) Guidelines, International Journal of Surgery. (2023) 109, no. 5, 1136–1140, 10.1097/js9.0000000000000373. 37013953 PMC10389401

